# Waves traveling over a map of visual space can ignite short-term predictions of sensory input

**DOI:** 10.1038/s41467-023-39076-2

**Published:** 2023-06-09

**Authors:** Gabriel B. Benigno, Roberto C. Budzinski, Zachary W. Davis, John H. Reynolds, Lyle Muller

**Affiliations:** 1 Department of Mathematics, Western University, London, ON Canada; 2 Brain and Mind Institute, Western University, London, ON Canada; 3 Western Academy for Advanced Research, Western University, London, ON Canada; 4 The Salk Institute for Biological Studies, La Jolla, CA USA

**Keywords:** Visual system, Neural encoding, Complex networks, Applied mathematics

## Abstract

Recent analyses have found waves of neural activity traveling across entire visual cortical areas in awake animals. These traveling waves modulate the excitability of local networks and perceptual sensitivity. The general computational role of these spatiotemporal patterns in the visual system, however, remains unclear. Here, we hypothesize that traveling waves endow the visual system with the capacity to predict complex and naturalistic inputs. We present a network model whose connections can be rapidly and efficiently trained to predict individual natural movies. After training, a few input frames from a movie trigger complex wave patterns that drive accurate predictions many frames into the future solely from the network’s connections. When the recurrent connections that drive waves are randomly shuffled, both traveling waves and the ability to predict are eliminated. These results suggest traveling waves may play an essential computational role in the visual system by embedding continuous spatiotemporal structures over spatial maps.

## Abstract (web summary)

Waves of neural activity travel across single regions in the visual cortex, but their computational role is unclear. Here, the authors present a neural network model demonstrating that waves traveling over retinotopic maps can enable short-term predictions of future inputs.

## Introduction

Five percent of synapses received by a neuron in the visual cortex arrive through the feedforward (FF) pathway that conveys sensory input from the eyes^1–4^. While these FF synapses are strong^5^, “horizontal” recurrent connections coming from within the cortical region make up about 80% of total synaptic inputs, with 95% of these connections arising from a very local patch (2 mm) around the cell^4^. The anatomy of the visual system thus indicates that cortical neurons interact with other neurons across the retinotopically organized maps^6^ that assign nearby points in visual space to nearby points in a cortical region via these horizontal connections. Models of the visual system predominantly focus only on FF^7,8^ and feedback (FB)^9^ connections. One result of this focus is that, in models of the visual system, neurons in the visual cortex are often modeled as non-interacting “feature detectors” with fixed selectivity to features in visual input (driven by FF connections) that can be modulated by expectations generated in higher visual areas (driven by FB connections). Neuroscientists have long been interested in how horizontal connections shape neuronal selectivity^10,11^ and “non-classical” receptive fields^12–16^. More recently, neuroscientists have also been interested in adding these connections to deep learning models to understand neuronal selectivity in the visual cortex^17,18^. It remains unclear, however, how horizontal connections shape the moment-by-moment computations in the cortex while processing visual input.

Recent analyses of large-scale recordings have revealed that horizontal connections profoundly shape spatiotemporal dynamics in the cortex. Traveling waves driven by horizontal connections have been observed in the visual cortex of anesthetized animals^19–24^. The relevance of traveling waves had previously been called into question, as they were thought to disappear in the awake state^25^ or to be suppressed by high-contrast visual stimuli^22,26^. Recent analyses of neural activity at the single-trial level, however, have revealed spontaneous^27^ and stimulus-evoked^28^ activity patterns that travel smoothly across entire cortical regions in awake, behaving primates during normal vision. These neural traveling waves (nTWs) shift the balance of excitation and inhibition as they propagate across the cortex, sparsely modulating spiking activity as they pass^29^. Because they drive fluctuations in neural excitability^27,30^, nTWs show that neurons at one point in a visual area (representing a small section of visual space) can strongly interact with neurons across the entire cortical region. These results thus indicate that cortical neurons may share information about visual scenes broadly across the retinotopic map through nTWs generated by horizontal connections.

What computations, then, can be done with waves of neural activity traveling across a map of visual space? To address this question, we studied a complex-valued neural network (cv-NN) processing visual inputs ranging from simple stimuli to natural movies. In these networks, activity at each node is described by a complex number. Complex numbers extend the arithmetic of the real number system, and as with standard, real-valued neural networks, nodes receive inputs based on connection weights, with the activity of each node determined by an activation function. The network state is then described by a vector of complex numbers, each element of which can represent the activation of a small patch of neurons in a single region of the visual cortex^31,32^. cv-NNs exhibit similar or superior performance to standard, real-valued neural networks in many supervised learning tasks^33^ and have been used effectively in explaining biological neural dynamics^34^. Here, we modified the standard FF architecture used in deep learning and computer vision to include horizontal recurrent connections, where neurons in a single processing layer form a web of interconnections similar to the horizontal connections in the visual cortex. Horizontal recurrent connections are thought to provide advantages^17^ over the standard FF architecture used in computer vision tasks^8,35^; however, current methods for incorporating recurrent horizontal fibers to convolutional network models of the visual system severely limit both the time window over which recurrent activity can be considered and the ease with which the networks can be trained^17^. In recent work, we have introduced a mathematical approach to understand the recurrent dynamics in a specific complex-valued model^36^. Here, we leverage this understanding to train recurrent complex-valued networks to process visual inputs, ranging from simple stimuli to naturalistic movie scenes. The resulting networks can predict learned movies many frames into the future, entirely from their internal dynamics alone, without external input. During prediction, the recurrent network exhibits prominent nTWs, ranging from simple waves propagating out from a small local input^28^ to complex traveling wave patterns^37^, raising the possibility that nTWs enable continuous predictions of dynamic and naturalistic visual input.

## Results

**Fig. 1:**
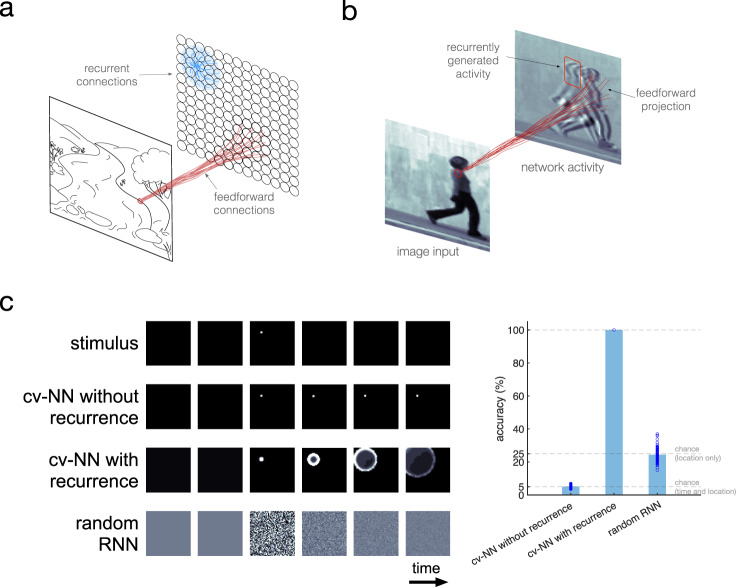
A topographic recurrent network model encodes spatiotemporal information of video frames via internal wave activity. **a** Schematic of the complex-valued neural network (cv-NN) model. Nodes (circles) are arranged on a two-dimensional grid and are recurrently connected (blue) locally in space like the cortical sheet. A natural image input projects locally into the network via feedforward connections (red), mimicking retinotopy. **b** Example dynamic of the network model. Due to the spatially local projection of the input image, an imprint of the image is visible in the grid of network activity. Due to the local recurrent connectivity, intrinsic wave activity is generated alongside the input projection. **c** Top row: In a sequence of six frames, exactly one of the first five contains a point stimulus, and the other frames do not. These frames are sequentially input to the network. Second row: When the cv-NN has no recurrence, the stimulus projection remains stationary. Third row: With recurrence, from the time of stimulus, cv-NN activity contains a projection of the stimulus and a wave radiating outward. Fourth row: Activity in a randomly connected recurrent neural network (RNN) following stimulus onset has a spatially disorganized structure, reflecting its lack of topography and distance-dependent time delays. Right: A linear classifier that received the final network state in the no-recurrence case could not predict the time or location beyond chance-level accuracy (5% overall), and in the random-RNN case, could predict the time but not the location beyond chance (25% overall). In contrast, using the classifier with the sixth with-recurrence network state allowed 100% accuracy since the feedforward projection of the point stimulus triggered a radiating wave that encoded the time and location of the stimulus in the subsequent network states. *N* = 100 trials for each group. Mean ± standard deviation of 5.09 ± 0.94, 100 ± 0, and 24.42 ± 4.13, respectively. Source data are provided as a Source Data file.

The cv-NN consists of an input layer sending movie frames to a recurrently connected neural network. An individual movie frame, serving as input to the network, is represented by a two-dimensional grid of pixels (input frame, Fig. 1a), and each pixel projects to the recurrently connected layer through FF connections (red lines, Fig. 1a). The recurrently connected layer is arranged on a two-dimensional grid, analogous to the retinotopic arrangement of neurons in visual regions. Horizontal interconnections within the cv-NN then drive recurrent interactions in the network (blue lines, Fig. 1a). Both FF and horizontal recurrent projections in the cv-NN are matched to the approximate scale of connectivity in visual cortex^38,39^ so that a single pixel in an input movie drives a local patch of neurons, with overlapping horizontal connections, in the cv-NN. Lastly, nodes in the recurrent layer communicate with time delays approximating axonal conduction speeds along horizontal fibers^40^, which have recently been shown to shape spiking neural activity into nTWs^29^. The combination of FF input and dense interconnections generates complex patterns of activity in the recurrent layer (Fig. 1b). Here, we focus on these recurrent activity patterns to understand their computational role for movie inputs ranging from simple to complex.

### nTWs can simultaneously encode stimulus position and time of onset over spatial maps

To illustrate how nTWs propagating over sensory maps could facilitate visual computation, we first studied the dynamics generated in response to a single point stimulus. Without recurrent connections, a short point stimulus generates a small bump of activity that remains centered on the point of input (“cv-NN without recurrence”, Fig. 1c). With recurrent connections, however, the point stimulus generates a wave that propagates out from the point of input (“cv-NN with recurrence”, Fig. 1c). We then studied these stimulus-evoked waves, which are similar in form to those previously observed in the visual cortex of awake primate^28^, in a simple decoding task. Specifically, we let the point stimulus appear at a random time and stimulus location in a series of input frames and then trained a linear classifier to decode the time and location of stimulus onset from the network activity at the final frame. As expected, in the cv-NN without recurrent connections, the classifier performed at chance-level accuracy in this task (Fig. 1c, right; “Methods”—“Stimulus prediction task”). With recurrence, however, the classifier selects the correct time and location of stimulus appearance from the final network state with 100% accuracy. Finally, while standard recurrent neural networks (RNNs) can encode time^41^, an RNN with random connections (and hence lacking the local connectivity and distance-dependent time delays in the cv-NN) also performs at chance level in this task, which requires decoding both stimulus location and onset time (Fig. 1c). This simple illustration shows that traveling waves of neural activity when propagating on an orderly retinotopic map can simultaneously encode stimulus location and onset time, even after the stimulus is no longer present.

### nTWs aid forecasting movie inputs from simple to complex

**Fig. 2:**
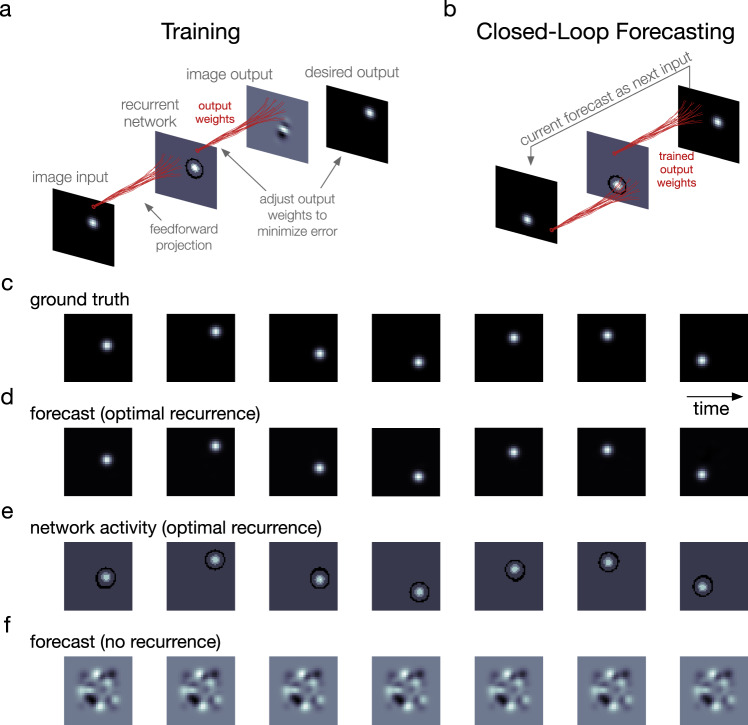
The network can forecast a simple video input many frames into the future. **a** As in the classification example (Fig. 1), a video frame projects into the network in a spatially local manner, and a recurrent network interaction occurs, generating internal wave activity on top of the projection. The network outputs an image from its network state via a matrix of trainable weights. Training entails one-shot linear regression between a set of network states and the corresponding desired output frames (the one-step-ahead next frames). Shown: a schematic representation of the one-shot linear regression for one time step. **b** Once training of the readout weights is complete, closed-loop forecasting begins. To properly test how well the network model learned the underlying spatiotemporal process from the training data, it is deprived of ground-truth data of any kind during this step. Instead, the forecast next frame at one time step serves as the input frame for the following time step. **c** Video frames of the data: a bump tracing an orbit. **d** Corresponding closed-loop forecasts generated by the network model with optimal recurrence. **e** Network activity for the optimal-recurrence case. Cosine of phase of activation is shown. **f** Closed-loop forecast in the case without recurrence.

Can nTWs enable the processing of the complex, dynamic, and nonstationary visual scenes that we encounter in our natural experience? We approached this question in several steps. We first asked whether, given an input frame from a movie, the cv-NN could be trained to accurately predict the following frame. To perform this more complicated task, we introduced a learning rule that requires training only a linear readout of the recurrent layer (Fig. 2a). This procedure is analogous to a complex-valued implementation of the reservoir computing paradigm^42^, which has recently found wide applications in nonlinear dynamics and physics. In the reservoir computing framework, an input signal drives activity in a recurrently connected layer. Activity in the recurrent layer is then decoded by a set of output weights, which are trained to produce a target output signal. Because of both its efficacy and relative efficiency in training, this framework has proven promising for learning predictive models of chaotic systems^43,44^, and reservoir computing has recently been used to learn and predict a range of important systems in physics^45,46^. This training process, however, has never before been applied to naturalistic movie scenes. We find the cv-NN can be reliably and efficiently trained to predict the next frame in a movie input (Supplementary Table 2, Moving Bump Input). With a cv-NN trained on a movie, the predicted next frame can then be provided as input in place of the original movie (Fig. 2b). Recent work on neural networks for processing movies has focused on predicting the next frame in a video sequence based on training on a large database of inputs^47–49^. In some cases, these predictions can then be fed back as input, allowing the network to recursively generate predictions from its own internal weights^50–58^. We will call this process, where during prediction, a network receives no external movie input and generates future predictions solely from its internal structure, *closed-loop forecasting* (CLF). Previous work has developed networks that can perform accurate CLF on the order of ten frames into the future^50–58^, with predicted frames becoming increasingly blurry. In this work, we asked a cv-NN to learn and perform CLF on individual movies. We find that cv-NNs trained on an individual movie can self-generate sharp forecasts of that movie many (25–100) frames into the future while receiving no external input. This system can be seen as a simple dynamical autoencoder, where a few input frames can ignite the self-generation of successive frames from its internal dynamics alone. This provides a framework that can give insight into how the visual system could create predictions by continuously changing weights based on its sensory input to make short-term extrapolations into the near future. The cv-NN is an effective model for closed-loop forecasting of entire visual scenes, generating accurate forecasts for movies of a few thousand pixels per frame using only a few thousand recurrently connected nodes.

The visual cortex readily processes and operates on dynamic visual inputs on timescales of milliseconds to seconds. We then asked whether closed-loop forecasting in this system could work on the scale of tens to hundreds of frames in an input movie. Starting with the first half of a movie containing a simple moving bump stimulus tracing out a trajectory in two-dimensional space (Fig. 2c), we find that the trained cv-NN can produce the entire second half of the movie as output from its trained synaptic weights alone (Fig. 2d and Supplementary Movie 1). As in the previous example, activity in the recurrent layer exhibits a dynamic spatiotemporal pattern extending beyond the immediate FF imprint of the stimulus and structured by the recurrent connections in the network (Fig. 2e and Supplementary Movie 1). These results demonstrate that recurrent cv-NNs can produce simple video inputs from their recurrent connections through this training process. Finally, when we remove the recurrent connections, the cv-NN produces an activity pattern that represents only the average of FF stimulus imprints without having learned the underlying spatiotemporal process^47^. In this case, the cv-NN no longer produces an accurate closed-loop forecast (Fig. 2f). These results demonstrate the importance of both the spatiotemporal patterns in the cv-NN and the horizontal recurrent dynamics generating them.

**Fig. 3:**
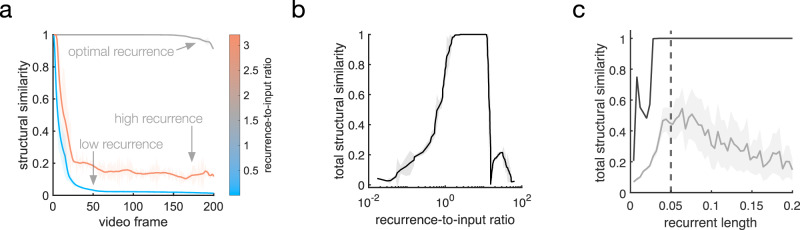
Moving bump forecast performance depends on specific properties of the recurrent connections. **a** Structural similarity (SSIM) between a forecast frame and the ground truth as a function of the closed-loop forecast video frame. Each curve corresponds to a different network parameter implementation. Curves have been smoothed by a moving-average filter (filter width of 30 time steps). Shaded error is the absolute difference between filtered and unfiltered. **b** Total structural similarity, in which a single SSIM is calculated for the whole movie as a function of the recurrence-to-input ratio. In the parameter space, each point differs only in recurrent strength. Smoothing and error shading is the same as in (**a**). **c** Total structural similarity as a function of recurrent length, which is the fraction of the network’s side length spanned by one standard deviation of the Gaussian connectivity kernel. In the three-dimensional parameter space comprising the recurrent strength (rs), recurrent length (rl), and input strength (is), averages (*n* = 89) across rs-is planes at fixed rl were computed (gray curve). Solid gray line: average. The peak coincides with the standard-deviation width of the Gaussian bump stimulus (dashed vertical line). Shaded area: variance. Solid black curve: maximum structural similarity at each recurrent length. Source data are provided as a Source Data file.

We find that closed-loop forecast performance in this system depends on two key factors: (1) the ratio of horizontal recurrent strength to feedforward input strength and (2) the spatial extent of the recurrence. To study the first factor in detail, we measured closed-loop forecast performance using an index of structural similarity (SSIM)^59^, which quantifies the perceptual match between two images, ranging between 0 (perfect mismatch) and 1 (perfect match). A threshold on the SSIM, determined through test comparisons between an original and noise-corrupted version of a movie, then provides a quantitative criterion for a successful closed-loop forecast (see Supplementary Fig. 2). We studied SSIM between movie frames produced by the closed-loop forecast process and the ground truth at different ratios of recurrence to input (Fig. 3a; see also Supplementary Fig. 1 and “Methods”—“Network connectivity” and “Network dynamics”). Once the stimulus is removed and the closed-loop forecast begins (video frame 1, Fig. 3a), forecast performance in cv-NNs with low recurrent strength quickly drops close to zero (light blue line, Fig. 3a). By contrast, cv-NNs at optimal recurrent strength sustain closed-loop forecasts for long timescales (gray line, Fig. 3a), extending beyond 100 video frames into the future. Importantly, networks where recurrence is too strong also perform poorly, with SSIM dropping near zero within a short timeframe (copper line, Fig. 3a). Systematic quantification of SSIM across ratios of recurrent strength to input strength reveals that performance is best when the recurrence and input are approximately balanced (Fig. 3b), in general agreement with the ratio of feedforward to recurrently generated synaptic drive in visual cortex^60,61^. We next studied performance as a function of the spatial extent of recurrent connectivity. The best performance occurs for recurrent lengths on approximately the same spatial scale as the moving bump stimulus (Fig. 3c), with performance dropping for recurrent lengths outside this range. This result demonstrates that recurrent connections aid closed-loop forecasting when matched to the spatial scale of the input. Horizontal recurrent connections in single visual regions span many different retinotopic scales^9,62^, which could enable processing stimuli at multiple spatial scales or moving stimuli with changing scales by the visual system.

**Fig. 4:**
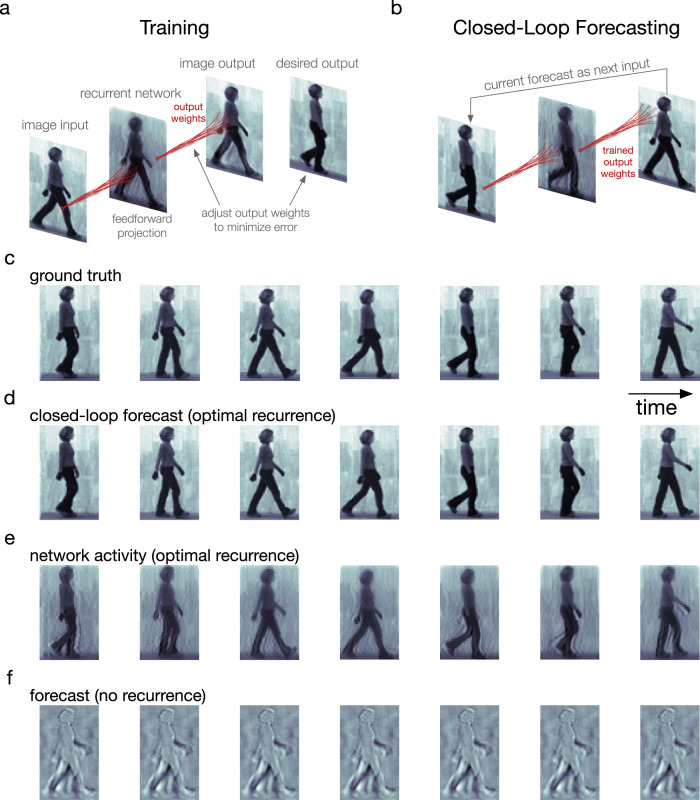
The recurrent network performs next-frame forecasting of a natural video input. **a** Training follows as in the moving bump example (Fig. 2a). **b** Next-frame closed-loop forecasting follows as in the moving bump example (Fig. 2b). **c** Video frames of the data: a person walking. **d** Corresponding closed-loop forecasts generated by the network model in the case of optimal recurrence. **e** Corresponding network states for the optimal-recurrence case (**d**). Cosine of phase is shown. **f** Same as (**d**), but in the absence of recurrence.

The visual system readily processes richly textured and naturalistic visual scenes. To examine this type of stimulus in the cv-NN, we considered naturalistic video inputs for next-frame prediction and closed-loop forecasting. To do this, we used videos from the Weizmann Human Action Dataset^63^. As above, we trained linear readout weights of the cv-NN on these individual naturalistic movie inputs (Fig. 4a) and then tested whether, given the first half of the input movie, the network could produce the second half in a closed-loop forecast (Fig. 4b). Even with a much more sophisticated input than the previous examples, the cv-NN can be trained rapidly and efficiently on the natural movie inputs (Supplementary Table 2, Walking Person Input). As in previous examples, at optimal values of the network parameters (“Methods”—“Parameter optimization”), the cv-NN accurately produces the natural movie using only its connection weights (Fig. 4c, d and Supplementary Movie 2). In this case, the recurrent connections in the cv-NN create complex wave patterns (Fig. 4e and Supplementary Movie 2). The recurrent connections and their resulting complex activity patterns are important for success in this task, as networks without recurrence do not produce accurate closed-loop forecasts (Fig. 4f).

**Fig. 5:**
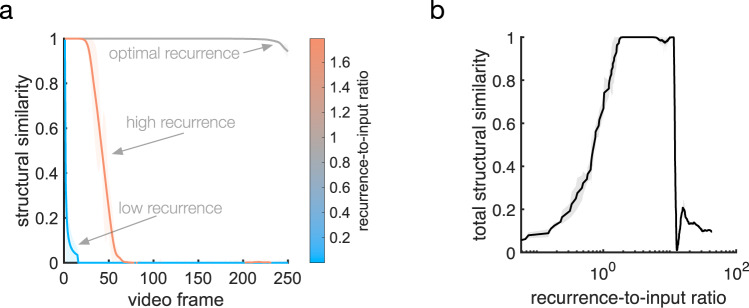
Natural movie forecast performance depends on specific properties of the recurrent connections. **a** Several examples of closed-loop forecast performance. Structural similarity (SSIM) between a forecast frame and the ground truth as a function of video frame during closed-loop forecasting. Each curve corresponds to a different ratio of recurrent strength to input strength. Curves have been smoothed by a moving-average filter (filter width of 30 time steps). Shaded error is the absolute difference between filtered and unfiltered. **b** Total structural similarity, in which a single SSIM is computed for the whole movie as a function of the recurrence-to-input ratio. In the parameter space, each point differs only in recurrent strength. Smoothing and error shading is the same as in (**a**). Source data are provided as a Source Data file.

**Fig. 6:**
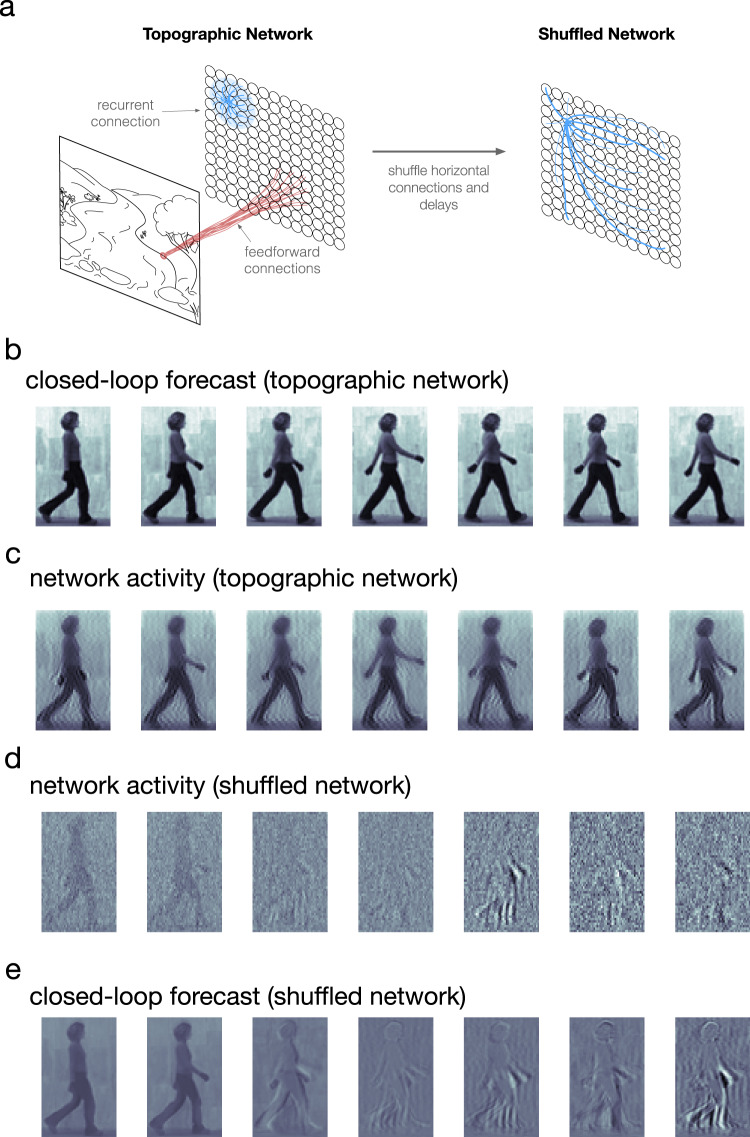
Randomly shuffling recurrent connections eliminates nTWs and the ability to forecast. **a** Left: the topographic network model used throughout this study, featuring feedforward projections of the image input (red lines) and local distance-dependent horizontal connectivity (blue lines). There are also synaptic time delays proportional to a node pair’s separation distance within the horizontal recurrent circuitry. Right: by randomizing the horizontal connection weights and time delays, the topography in the network is removed. **b** Closed-loop forecasts generated by the topographic network. **c** The network activity of the topographic network in response to frames of a natural movie input. **d** Network activity of the shuffled network. **e** Closed-loop forecasts generated by the shuffled network.

We then studied what specific features of the recurrent connections enable predicting naturalistic movie inputs. As in the moving bump example, networks perform best when recurrence and input are approximately balanced, and the performance quickly decays when the recurrence is too weak or too strong (Fig. 5a, b). This result shows that, as in the simple case of the moving bump, the complex spatiotemporal predictions generated by the network depend on a sophisticated interplay between input and recurrent connections. We next studied the role of connection topography and distance-dependent time delays. To do this, we started with networks that achieve accurate predictions and randomly shuffled both the connections and time delays, a control that removes the two key factors for generating nTWs in large-scale spiking network models^29^ that match waves observed in the visual cortex (Fig. 6a). We then compared the closed-loop forecast performance and network activity in the topographic and shuffled cases. In the topographic case, the cv-NN produces accurate predictions and complex traveling wave patterns, as before (Fig. 6b, c). The shuffled versions of the cv-NN, however, produce spatiotemporally unstructured activity in the recurrent layer (Fig. 6d) and do not achieve accurate closed-loop forecasts, even after the cv-NN was retrained (Fig. 6e; see also Supplementary Table 3 and Supplementary Movie 3). This result demonstrates that with all other architectural features of the network held constant, a randomly connected cv-NN that does not produce nTWs cannot be trained to perform CLF using the same procedure that was previously successful. Shuffling only time delays in the cv-NN and then retraining also substantially drops closed-loop forecast performance (decreasing total structural similarity from 0.99 to 0.02). Further, reducing the conduction speed in half and then retraining also results in a substantial drop in performance (from 0.99 to 0.08). These two control analyses demonstrate that successful closed-loop forecasts depend on a range of time delays in the cv-NN. Finally, the specific spatiotemporal structure of the input movie is also important: a cv-NN at the optimal hyperparameters for a natural movie cannot be retrained to do closed-loop forecasting on a randomized (phase-shuffled) version of the same movie (Supplementary Table 1), demonstrating that the cv-NN utilizes the specific spatiotemporal correlations in the movie to generate its forecast. Taken together, these results demonstrate that the complex spatiotemporal patterns generated by horizontal recurrent connections in the cv-NN enable performance on next-frame prediction and closed-loop forecasting tasks for sophisticated natural movie inputs.

### The nTW network model is capable of forecasting multiple movies without retraining

**Fig. 7:**
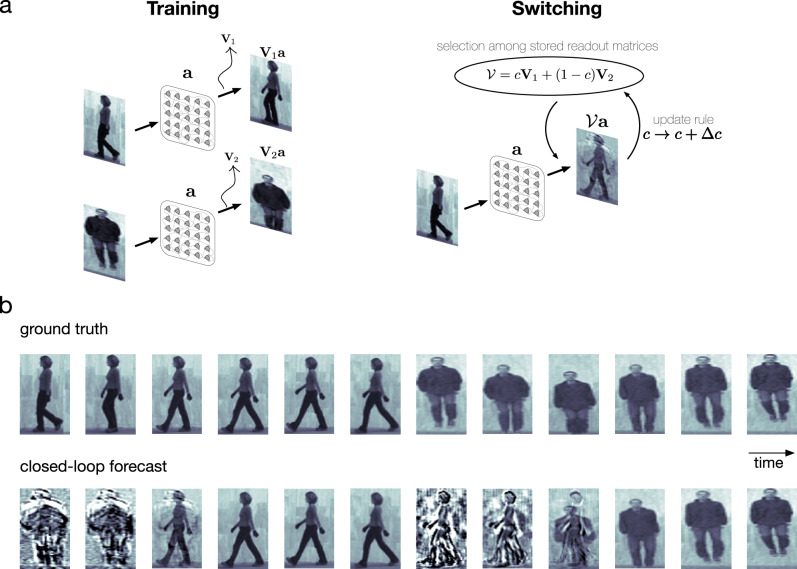
The network is capable of forecasting multiple movies without being retrained. **a** The recurrent network model was adapted to contain a higher-level competitive-learning process. Left: Readout matrices were learned separately for separate examples. Right: Storing the learned readout matrices in an aggregate matrix ${{\mathcal{V}}}$, the present network state drove the aggregate matrix toward either of the learned matrices via an unsupervised competitive learning rule. **b** Beginning with feeding frames from movie 1, the network takes some time to recall the learned matrix that results in an accurate closed-loop forecast. Quickly switching to a different movie, the network once again takes some time to adjust its output weights before converging to the correct ones for an accurate closed-loop forecast.

We lastly sought to understand whether the cv-NN could perform closed-loop forecasts on multiple movies it had previously learned and switch flexibly with changing inputs. To do this, we implemented a simple competitive process (“Methods”—“Movie switching”) so that the network could adapt its output based on the similarity of its prediction to its input (Fig. 7a). Specifically, output weights for the cv-NN were trained on individual movies (${{\mathbf{V}}}_1$ and ${{\mathbf{V}}}_2$, cf. “Training” in Fig. 7a) and stored in an aggregate matrix (${{\mathcal{V}}}$, cf. “Switching” in Fig. 7a). When performing a closed-loop forecast, this extended network model can receive new input from this previously learned set, and then rapidly switch to closed-loop forecasting this new movie input within a few frames without any retraining of weights in the individual output matrices ${{\mathbf{V}}}_i$ (Fig. 7b and Supplementary Movie 4). This result demonstrates that the process of closed-loop forecasting, mediated by horizontal recurrent fibers in the network, can generalize to realistic visual conditions with multiple, changing input streams.

## Discussion

In this work, we have introduced a model to understand whether traveling waves generated by horizontal connections in the visual cortex may play a computational role in processing natural visual inputs. By adapting a recurrent neural network model using a specific dynamical update rule and learning rule, this model learns to forecast video inputs ranging from simple visual stimuli to complex natural scenes. We report here a network model that can be trained to produce quantitatively verified closed-loop forecasts of richly textured naturalistic movies many frames into the future. The cv-NN introduced in this work incorporates the spatial topography and time delays important for shaping activity dynamics in single regions of the visual system^29^ and provides a potential computational role for waves of neural activity traveling over maps of visual space. Whether similar principles of spatial topography could benefit RNNs, in general, remains open but represents an interesting potential direction for future work. Further, because the recurrent dynamics in the cv-NN are tractable to detailed mathematical analysis^36^, this recurrent network model opens new possibilities for understanding the mechanisms underlying successful predictions studied here and for designing new applications in future work.

Closed-loop forecasting in the cv-NN demonstrates a form of short-term prediction by nTWs that may be relevant to the online processing of continuous sensory input by the visual system. Consider, for example, a batter in the game of baseball facing a pitcher who has just pitched a curveball, now hurtling toward the batter at over 100 miles per hour. In major league baseball, a pitch takes around 400 milliseconds to travel 60 feet from the pitcher’s hand to the batter at home plate. Time is required for the neural computations that enable the batter to perceive the ball and estimate its trajectory. This includes both the time required for sensory information to travel from the retinae to relevant brain areas and the time required for computation of the ball’s trajectory in space based on these signals. Assuming the entire computation can be accomplished in 150 milliseconds^64^, during this time, the ball will have traveled more than 22 feet. To estimate the likely current location of the ball based on information that was available to the visual system 150 milliseconds ago, the brain may form an internal model of the ball’s trajectory in space, informed by previous experience. Consistent with this idea, batters often report that, as the spinning ball travels from the pitcher’s mound to home plate, the curveball suddenly changes direction, an illusory percept referred to as the curveball’s “break”^65^. Short-term predictions by nTWs may represent one mechanism for rapid estimation of trajectories, as continuous spatiotemporal structures propagating over the retinotopic map. In this way, closed-loop forecasts in the cv-NN could enable the visual system to estimate the likely trajectory of the ball based on training from the previous visual experience. The curveball’s “break” further recalls the process of switching predictions when the input becomes sufficiently discrepant with incoming sensory data (Fig. 7b). When the movie switches from one input to another (top row, “ground truth”), the network generates a transiently indeterminate activity pattern before jumping to the correct forecast (bottom row, “closed-loop forecast”). In this way, the cv-NN may provide a mechanistic framework for specific hypotheses in future work about the interaction of short-term predictions generated by recurrent horizontal fibers and continuously incoming sensory input. The cv-NN could also be useful as a model to explain how the brain encodes, stores, and recovers episodic memories of richly textured visual scenes, which studies of visual search^66^ and vivid recollection^67,68^ have shown are associated with activity in visual regions.

Further, while the cv-NN is not intended to be a veridical simulation of the millions of neurons contributing to nTW dynamics in the visual cortex, this network model is broadly consistent with spatiotemporal dynamics recently observed in the visual system of the alert primate. In the case of a single point stimulus (Fig. 1c), the network produces a traveling wave radiating out from the point of input. This is similar to nTWs detected in single trials during voltage-sensitive dye optical imaging in the primary visual cortex (V1) of awake macaques^28^. nTWs evoked by small visual stimuli (Gaussian spot, with a standard deviation of 0.5° of visual angle) presented during fixation consistently evoked nTWs that propagate over 7.5 mm of V1, representing a significant portion of this cortical area^69^. The spatial extent of the nTWs observed in the experiment provides a point of comparison with the model, as spatial extent determines the scale at which local populations in V1 may influence others across the retinotopic map. In the cv-NN, waves generated by small point stimuli propagate over slightly more than one-third of the network (decaying to half-amplitude after traveling over 37.5% of the network; Fig. 1c). These results demonstrate that nTWs may propagate over broadly similar spatial extents in visual cortex and in the cv-NN.

Another point of comparison with measured neural dynamics centers on the patterns evoked by moving stimuli. In the case of a moving bump stimulus (Fig. 2), the network produces a bump of activity, reflecting FF input driven by the movie but also reflecting recurrently generated activity that extends beyond the feedforward imprint of the stimulus (Fig. 2e). The radius of this recurrently generated activity is approximately twice that of the feedforward bump. This result recalls analyses of Utah array recordings in V1 of awake macaques^70^. Using a moving bar stimulus (0.5 × 4° of visual angle, moving horizontally at 6.6° per second), the authors found responses in V1 before stimuli entered neurons’ classical receptive field (cf. Fig. 2C in ref. ^70^). The onset times of these anticipatory responses became earlier and earlier along the moving bar’s trajectory. These changes in time were confirmed with computational analyses and modeling to be consistent with propagation along horizontal fibers in V1, and the spatial extent of the recurrent interactions is, again, approximately consistent with dynamics during closed-loop forecasting in the cv-NN.

The dynamics of the cv-NN are thus broadly consistent with observations of neuronal dynamics during normal processing in awake, behaving primates. Recent work has demonstrated the importance of the topographic connection patterns and axonal time delays matching those found in the visual cortex to generate nTWs in large-scale spiking network models^29^. Recent theoretical studies have developed complex-valued network models that can provide analytical insight into the time-varying dynamics of spiking neural networks^31,71^, and future work could directly relate dynamics in the cv-NN during movie prediction to the fine-scale spiking dynamics of the networks in the visual cortex. Finally, in the case of naturalistic movie inputs (Fig. 4), the cv-NN produces complex spatiotemporal patterns that can be mathematically described in this model as the summation of multiple traveling waves^36,37^. Future work analyzing large-scale recordings will provide opportunities for comparison between activity patterns in the visual cortex and in the cv-NN during the processing of naturalistic movie inputs.

Another potential extension of the cv-NN is to consider multiple recurrently connected layers with specializations similar to those in different regions of the visual cortex. In this work, we focused on a cv-NN with a single recurrently connected layer to understand the potential computational role of nTWs that have recently been observed in single cortical regions during visual perception in awake animals. nTWs have been observed in many visual areas, including V1^28^, V2^28^, V4^72^, and MT^24,27,73^. Adding multiple recurrent layers in the cv-NN may provide opportunities in future work for understanding nTW dynamics across visual areas, where spatiotemporal activity patterns have recently been shown to propagate in feedforward and feedback directions in different frequency ranges^74^. Finally, closed-loop forecasts in this cv-NN are not intended to be robust to arbitrary translations or rotations of the visual scene, and adding multiple layers in the cv-NN may provide a degree of translation invariance, which is achieved in CNNs through cascading activity through multiple processing layers^75^, and scale invariance, which may also be made possible through processing in multiple recurrent layers^76^. In this way, extending the cv-NN with multiple recurrent layers represents an important opportunity for understanding the organization and computational role of nTWs occurring in many cortical areas in future work.

These results provide fundamental insight into the function of horizontal recurrent connections, whose effect on the moment-by-moment computations in the visual system has remained unexplained. While there has been much interest in the function of recurrent horizontal fibers in the visual cortex, for example, in explaining direction and orientation selectivity in V1^10,11^ or in center-surround models of the receptive field^14,16,77^, general computational roles for traveling waves generated by the massive recurrent circuitry in single cortical areas on the single-trial level remain unknown. Successful models of the visual system, including feature-based models and deep convolutional neural networks, have provided insight into how neural systems could process single image inputs but explain only a fraction of the variance in neural responses to natural sensory stimuli^18,78,79^. Importantly, it is not necessarily the case that all RNNs that can perform CLF will also exhibit nTWs; however, when networks possess the main architectural features found in the visual cortex (local connections, retinotopically ordered inputs, and communication time delays), we have demonstrated that nTWs are tightly linked to CLF. The cv-NN may thus provide new opportunities for understanding how the visual system processes continuously updated, movie-like visual inputs, where information is extracted from the visual environment moment-by-moment as it comes from the eye. The sophisticated closed-loop movie forecasts produced by this network, and the fact that this closed-loop forecast process can generalize to multiple movie inputs, represent an important step in explaining the computational role of recurrent connections and traveling waves in the visual cortex.

## Methods

Custom MATLAB (version R2021a) code was used for all data simulation and analysis in this study.

### Network connectivity

The recurrent network is arranged on a square grid of $N$ nodes. The network grid is treated as a discretized Euclidean plane such that the side lengths span distances of unity. Boundaries are not periodic. The recurrent weight ${w}_{{ij}}$ from node $j$ to node $i$ is inversely proportional to their Euclidean distance ${d}_{{ij}}$ so as to give local connectivity like that of the neocortical sheet. Specifically, ${w}_{{ij}}$ is Gaussian as a function of ${d}_{{ij}}$:

1 $${w}_{{ij}}=\alpha \,{{\exp }}\left[-{d}_{{ij}}^{2}/(2{\beta }^{2})\right].$$

The coefficient $\alpha$ is called the recurrent strength, and the standard deviation $\beta$ is called the recurrent length. Both are free parameters. The maximum possible value of ${d}_{{ij}}$ is $\sqrt{2}$ (corner to corner), and, for example, $\beta=1$ means that the recurrent length equals the network side length. Further, all ${N}^{2}$ such weights are strictly positive, and the $N$-by-$N$ matrix of such weights is symmetric (${w}_{{ij}}={w}_{{ji}}$). Diagonal weights (${w}_{{ii}}$) are not set to zero.

### Network dynamics

Network dynamics are given by a complex-valued equation. A complex number $z$ is of the form $z=x+{{{\mathrm{i}}}}y$, where $x$ is the real part, $y$ is the imaginary part, and ${{\mathrm{i}}}$ is the imaginary constant defined as ${{{\mathrm{i}}}}^{2}=-\!1$. Equivalently, $z = m \exp[{\mathrm {i}}{\phi}]$, where $m$ is the modulus and $\phi$ is the argument. A complex number is intuitively visualized as a two-dimensional vector, where $\left(x,y\right)$ is its Cartesian representation and $\left(m,\phi \right)$ is its polar representation. What distinguishes a complex number from a standard two-dimensional vector is the multiplication rule: multiplication of two complex numbers corresponds to both a scaling and a rotation in the so-called complex plane. This property makes complex-valued representations of observable quantities more concise than real-valued representations, and thus, complex numbers are a central tool in physics and engineering. From the perspective of biological vision, a complex-valued representation is useful. Since phase information is important for representing visual inputs, complex-valued models, which efficiently represent phase in the argument $\phi$, are ideal. Indeed, complex-valued models of vision are widely explored^80^. Given the practical utility of artificial neural networks and deep learning (including for modeling biological neural networks), complex-valued neural networks, in which the neural activations are complex-valued, are of great interest. However, they are notoriously difficult to train, especially in a recurrent architecture^32^. We make an advance here on this front by choosing a unique dynamical equation and by exploiting the advantages of reservoir computing.

The discrete-time dynamical equation for each node $i$ is

2 $${a}_{i}[t+1]={a}_{i}[t]+{x}_{i}[t]-{{{\mathrm{i}}}}\mathop{\sum }\limits_{j=1}^{N}{w}_{ij}\exp \left\{{{{\mathrm{i}}}}({a}_{j}[t-{\tau }_{ij}]-{a}_{i}[t])\right\},$$

3 $${a}_{i}[t+1] \, := {a}_{i}[t+1]/\left|{a}_{i}[t+1]\right|.$$

Here, ${a}_{i}[t]$ is the complex-valued activation at discrete time $t$, ${x}_{i}[t]$ is the feedforward input of the image stimulus to node $i$ at discrete time $t$, and ${w}_{{ij}}$ is the recurrent weight from node $j$ to node $i$ (“Methods”—“Network connectivity”). Further, ${\tau }_{{ij}}$ is the discrete time delay between nodes $i$ and $j$, given by ${\tau }_{{ij}}=$ round$[{d}_{{ij}}/v]$ in which the Euclidean distance ${d}_{{ij}}$ between nodes $i$ and $j$ (“Methods”—“Network connectivity”) is scaled by the parameter $v$, which represents the speed of activation transmission across the network, and round$[{d}_{{ij}}/v]$ rounds ${d}_{{ij}}/v$ to the nearest integer in accord with the discrete-time dynamics. A $v$-value of, for example, $v=0.1$ means the activation travels a distance of one-tenth the network side length per time step. Lastly, the modulus of ${a}_{i}[t]$ (i.e., $\left|{a}_{i}[t]\right|$) is normalized (Eq. (3)), which confines ${a}_{i}[t]$ on the complex unit circle, and thus, the phase of ${a}_{i}[t]$ contains the dynamics. We note that modulus normalization is a common operation used in complex-valued neural networks^32^.

The specific form of Eq. (2) is unique compared to other complex-valued neural-network equations because it involves a pairwise node attraction ${a}_{j}[t-{\tau }_{{ij}}]\,-\,{a}_{i}[t]$. Another system with pairwise attraction is the Kuramoto model, a popular model for studying synchronization in nonlinear systems^81–83^. Our presented system has a correspondence with the Kuramoto model^84^ and allows the description of the dynamics for individual realization in terms of the eigenvalues and eigenvectors of the network^36^. With the described local network connectivity and distance-dependent delays, the presented system gives rise to meaningful spatiotemporal self-organization dynamics.

The initial network state is ${a}_{i}[0]=0+0i$ for all nodes, and the first several time steps contain transient activity associated with the input disrupting the initial steady state of the system. For the stimulus prediction task, this transient activity is important to the model and was used, while for the next-frame forecasting task, it is distracting to the model and was discarded.

### Image read-in

At each discrete time step, a digital grayscale image is read into the network. Prior to read-in, the image is mean-subtracted and divided by its standard deviation across all its pixels (i.e., *z*-scored). Image read-in is accomplished with a local feedforward projection, which mimics retinotopy and preserves the spatial correlations in the image. Technically, this is a two-dimensional interpolation using the bilinear kernel common in image processing, which takes a weighted average in the nearest 2-by-2 pixel neighborhood. The projected image has $\sqrt{N}$ rows and $\sqrt{N}$ columns like the network grid, and each pixel intensity of the projected image is given by ${x}_{i}[t]$ (Eq. 2). Lastly, ${x}_{i}[t]$ is scaled according to ${x}_{i}[t]\,:=\gamma {x}_{i}[t]$, where $\gamma$ is called the input strength. In our model, $\gamma$ is the fourth and final free parameter after the recurrent strength, recurrent length, and conduction speed.

### Stimulus prediction task

The classification was performed using the basic perceptron. For an input vector **v** $={\left[1\,{v}_{1}\,\cdots {v}_{N}\,\right]}^{T}$, where ${v}_{1},\,\ldots,\,{v}_{N}$ are features, and a label $l\in \{{{{{\mathrm{0,1}}}}}\}$, the goal is to find a hyperplane **u**^**T**^**v**$=b+{u}_{1}{v}_{1}+\cdots+{u}_{N}{v}_{N}=0$, where **u** $={\left[b\,{u}_{1}\,\cdots\,{u}_{N}\right]}^{T}$ is a vector containing the bias $b$ and weights ${u}_{1},\,\ldots,\,{u}_{N}$, that separates the data in the $N$-dimensional feature space according to their binary class (0 or 1). During training, with a sub-optimal **u-**vector and one example **v**-vector, the output classification $l={\mathrm {H}}$(**u**^**T**^**v**) is computed, where H($\cdot$) is the Heaviside step function defined as unity for positive argument and zero otherwise. For the desired classification $d$ (either 0 or 1), the signed distance $\varDelta=d-l$ is computed, where $\varDelta \in \left\{-{{{{\mathrm{1,0,1}}}}}\right\}$. With each new example **v**, the **u-**vector is updated using the delta rule **u **:= **u** + $\lambda$**v**$\varDelta$, where $\lambda$ is the learning rate. To use the perceptron in multiclass classification, the one-versus-rest scheme is used. That is, for the set of classes $C=\left\{{c}_{1},\ldots,\,{c}_{M}\right\}$, binary classification is performed separately $M$ times. Each time $i$, the two classes are defined such that ${c}_{i}=1$ and ${C\backslash }{c}_{i}=0$, where “$\backslash$” denotes the set difference. Then, there are $M$ weight vectors **u**_1_,…, **u**_M_, and $M$ inner products ${f}_{1}=$**u**_1_^**T**^**v**, …, ${f}_{M}=$**u**_M_^**T**^**v** for a given data vector **v**. The multiclass classification is $\mathop{\mathrm {argmax}}\limits_{{c}_{i}} \left[{f}_{1},\,\ldots,\,{f}_{M}\right]$.

In the stimulus classification task (Fig. 1c), input frames were 50 by 50 pixels, and the network was 50 by 50 nodes. There were six frames. One of the first five frames was randomly chosen to contain the point stimulus, and the remaining frames were entirely zero intensity. The point stimulus was an isotropic two-dimensional Gaussian of standard deviation of 0.05, and the input frames are defined on the Cartesian grid $\left[-{{{{\mathrm{2,2}}}}}\right]\times \left[-{{{{\mathrm{2,2}}}}}\right]$. The stimulus was centered in one of four equally sized quadrants in the frame. The sequence of frames was sequentially input to the network. There are exactly twenty classes: each of the first five frames times each of the four quadrants in which the point stimulus could occur. The column vector of activations corresponding to the final (sixth) frame was used as predictor for all trials. The task was repeated 100,000 times, with the time of stimulus (1 or 2 or 3 or 4 or 5) and the location of the stimulus (quadrant 1 or 2 or 3 or 4) randomly rechosen each time.

### Closed-loop forecasting

The network outputs an image of ${M}_{r}$ rows and ${M}_{c}$ columns of pixels—the same size as the input image—at each time step. In both examples (moving bump and natural movie), the network was 50 by 50 nodes ($N=2500$). Recalling that ${a}_{i}[t]$ is the complex-valued activation of node $i$ at discrete time $t$ (Eqs. (2) and (3)), the output transformation is linear:

4 $${y}_{i}[t]={\sum }_{j=1}^{N}{v}_{{ij}}{a}_{j}[t]^{\prime} .$$

Here, ${y}_{i}[t]$ is the $i$^th^ pixel intensity of the output image, and ${v}_{{ij}}$ is the $(i,j)$^th^ readout weight of the $M$-by-$N$ matrix **V**, where $M={M}_{r}{M}_{c}$. The prime notation (′) indicates that the activation vector **a**[t]$={\left[{a}_{1}[t]\cdots {a}_{N}[t]\right]}^{T}$ was mean-subtracted, which was done to avoid an intercept term during training.

The readout weights $\left\{{v}_{{ij}}\right\}$ of **V** are the only weights trained in our model, making our network a reservoir computer. Reservoir computers are recurrent neural networks that avoid the issues associated with training recurrent weights and have been shown to perform well in time series forecasting^42^. Suppose training begins at time step 1, after discarding the initial transient, and ends at time step T. Defining **a**[t]′ $={\left[{a}_{1}[t]^{\prime} \cdots {a}_{N}[t]^{\prime} \right]}^{T}$, the matrix of regressors is then

5 $${{{{{\bf{A}}}}}}=\big[{{{{{\bf{a}}}}}}[1]^{\prime}\ldots {{{{{\bf{a}}}}}}[{{{{{\rm{T}}}}}}]^{\prime}\big]$$

and the matrix of regressands (desired outputs) is

6 $${{{{{\bf{D}}}}}}=\big[{{{{{\bf{f}}}}}}[2]\ldots {{{{{\bf{f}}}}}}[{{{{{\rm{T+1}}}}}}]\big].$$

Hence, the desired outputs are simply the set of one-step-ahead frames. Here, **f**[t] is the column vectorization of the $t$^th^ input image frame (before read-in) and is also mean-subtracted. Training entails ordinary least-squares linear regression between **A** and **D**. Because **D** is highly underdetermined (containing far fewer frames than pixels per frame), the matrix 2-norm of **V** was simultaneously minimized during regression to reduce model bias.

Following training is *closed-loop forecasting*. At this point, the network activation has been primed by being driven with the training frames, and the readout matrix **V** has been trained. In the first time step of closed-loop forecasting, we input the corresponding video frame. Subsequently, for steps $\{t\}$, the predicted output at time step $t$ serves as the input for time step $t+1$.

In the moving bump example (Fig. 2), the frames are 30 by 30 pixels and defined on a $\left[-{{{{\mathrm{2,2}}}}}\right]\times \left[-{{{{\mathrm{2,2}}}}}\right]$ Cartesian grid. A two-dimensional isotropic Gaussian of standard deviation 0.2 traced a Lissajous curve given by the parametric equations ${x}_{c}(t)=\sin (t/3)$ and ${y}_{c}(t)=\cos (t/3)$, where $({x}_{c},{y}_{c})$ is the center of the Gaussian in space and $t$ is a continuously valued time variable^85^. The Lissajous trajectory was discretized to have 100 frames per cycle. The first cycle was discarded to omit the initial transient network activity, the network was trained on the subsequent 3 cycles, and closed-loop forecasting was performed on the 2 cycles subsequent to that.

In the natural video example (Fig. 4), a walking video from the Weizmann Human Action Dataset^86^ was used, in which a person walks across the scene. We present several key examples here but note that the model successfully performs closed-loop forecasting for all movies in this dataset, where we define a successful closed-loop forecast as one in which the total structural similarity is at least 0.9 (Supplementary Fig. 2, Supplementary Table 4). Segmentation masks of the people in the videos are included with this dataset (https://www.wisdom.weizmann.ac.il/~vision/SpaceTimeActions.html). Using these masks, we cropped the frames so that the person was centered throughout the entire walk, giving frames of approximately 80 by 50 pixels. Without performing this step, our network model would fail: the training data would be independent of the closed-loop forecast data since they would occupy exclusive regions of the pixel space, and the model would not generalize to the prediction data. Such nonstationary data have been successfully taught to networks with approximate translation invariance, and translation invariance is likely used in the brain to learn such processes^87^. However, translation invariance is beyond the scope of our study. The frames were then resized to be exactly 80 by 50 pixels. Finally, each video was around 70 frames long. To get more frames without interpolation, we “bookended” each video by concatenating it with its temporal reverse sequence, where one cycle consists of the original frames followed by the bookended frames. The result is a longer video with the same spatiotemporal statistics. The first cycle was discarded to omit the initial transient network activity, the network was trained on the subsequent three cycles, and the closed-loop prediction was performed on the two cycles subsequent to that.

To measure the balance between feedforward input and recurrent interaction, we devised the *recurrence-to-input ratio*. Per Eq. (2), the input and recurrence terms are the column vectors **x**[t] $={\left[{x}_{1}[t]\cdots {x}_{N}[t]\right]}^{T}$ and **r**[t]$={\left[{r}_{1}[t]\cdots {r}_{N}[t]\right]}^{T}$, respectively, where

7 $${r}_{i}[t]=-{{{\mathrm{i}}}}\mathop{\sum }\limits_{j=1}^{N}{w}_{ij}\exp \left\{{{{\mathrm{i}}}}({a}_{j}[t-{\tau}_{ij}]-{a}_{i}[t])\right\}.$$

Further, let the matrices

8 $${{{{{\bf{R}}}}}}=\big[{{{{{\bf{r}}}}}}[1]\ldots {{{{{\bf{r}}}}}}[{{{{{\rm{T}}}}}}]\big]$$

and

9 $${{{{{\bf{X}}}}}}=\big[{{{{{\bf{x}}}}}}[1]\ldots {{{{{\bf{x}}}}}}[{{{{{\rm{T}}}}}}]\big]$$

be the horizontal concatenations of **r**[t] and **x**[t], respectively, over closed-loop forecast times $\left\{t,{t}+1,\,\ldots,{t{{\hbox{'}}}}\right\}$. The ratio is defined as

10 $${|{{{{{\bf{R}}}}}}|}_{{{{{{\rm{F}}}}}}}/{|{{{{{\bf{X}}}}}}|}_{{{{{{\rm{F}}}}}}},$$

where ||**G**||_F_ denotes the Frobenius matrix norm of a matrix **G**, which is equivalent to the Euclidean vector norm of the vectorization of **G**.

### Movie switching

The network was trained on two movie inputs: one of a walking person (movie 1) and one of a jumping person (movie 1), both from the Weizmann dataset. The same recurrent matrix was used in each case–only the learned matrices (**V**_1_ and **V**_2_, respectively) differed. Let ${{\mathcal{V}}}$  = c**V**_1_ + (1−c)**V**_2_, where $c\in [{{{{\mathrm{0,1}}}}}]$. ***v*** stores both learned matrices, and the present input modulates the relative contribution of **V**_1_ and **V**_2_ using an update rule for $c$. The structural similarity between the input and output were computed at each time step t (S$[t]$), and the change thereof was computed at each time step as $\varDelta$S = S[t] − S[t−1]. The update rule is $c := c+\Delta c$, where ∆c = −*η*sgn[*Δ*S] and $\eta$ is the learning rate, set to 0.1. Depending on which movie (movie 1 or movie 2) drives the network, $c$ tends toward 1 or 0, respectively. Once this happens, this driving input is removed and closed-loop forecasting commences as described. Switching entails instantaneously transitioning from closed-loop forecasting of one movie to driving the network with the frames of another movie. $c$ then updates as described and is followed by closed-loop forecasting again.

### Parameter optimization

The random-search algorithm was used to optimize parameters for closed-loop forecasting. Within specified bounds, each parameter was randomly sampled, giving a point in the parameter space. The parameter space was randomly sampled in this way many times, and each time, the structural similarity index was computed as the performance index. The bounds within which the parameters were sampled are given in Table 1.

**Table 1:** Intervals over which model parameters were randomly searched during optimization

Parameter	Sampled interval
Recurrent strength	(0, 0.2)
Recurrent length	(0, 0.2)
Input strength	(0, 0.2)
v	(0, 0.1)

### Reporting summary

Further information on research design is available in the Nature Portfolio Reporting Summary linked to this article.

## Supplementary information

Supplementary Information

Description of Additional Supplementary Files

Supplementary Movie 1

Supplementary Movie 2

Supplementary Movie 3

Supplementary Movie 4

Reporting Summary

## Data Availability

The point stimulus and moving bump stimulus data generated in this study can be generated from the code available at this study’s GitHub repository (https://github.com/mullerlab/benignoEAwavecomp). The raw data of the natural movies used in this study are provided by Lena Gorelick, Moshe Blank, and Eli Shectman of the Weizmann Institute of Science, available at https://www.wisdom.weizmann.ac.il/~vision/SpaceTimeActions.html and this study’s GitHub repository. Source data are provided with this paper.

## Source data

Source Data

## Code availability

All codes associated with this study are available at https://github.com/mullerlab/benignoEAwavecomp^88^.
