# Supplementary material for: Waves traveling over a map of visual space can ignite short-term predictions of sensory input
Source: Nat Commun. 2023 Jun 9;14:3409. doi: 10.1038/s41467-023-39076-2 (PMC10256723; doi:10.1038/s41467-023-39076-2)
Supplement: Supplementary file 1 — Supplementary Information [file 41467_2023_39076_MOESM1_ESM.pdf]

Supplementary Information for "Waves traveling over a map of visual space can ignite short-term predictions of sensory input"

Gabriel B. Benigno,[1,2,3] Roberto C. Budzinski,[1,2,3] Zachary Davis,[4] John Reynolds,[4] and Lyle Muller[1,2,3]

[1] *Department of Mathematics, Western University, London, ON, Canada*
[2] *Western Institute for Neuroscience, Western University, London, ON, Canada*
[3] *Western Academy for Advanced Research, Western University, London, ON, Canada*
[4] *The Salk Institute for Biological Studies, La Jolla, CA, USA*

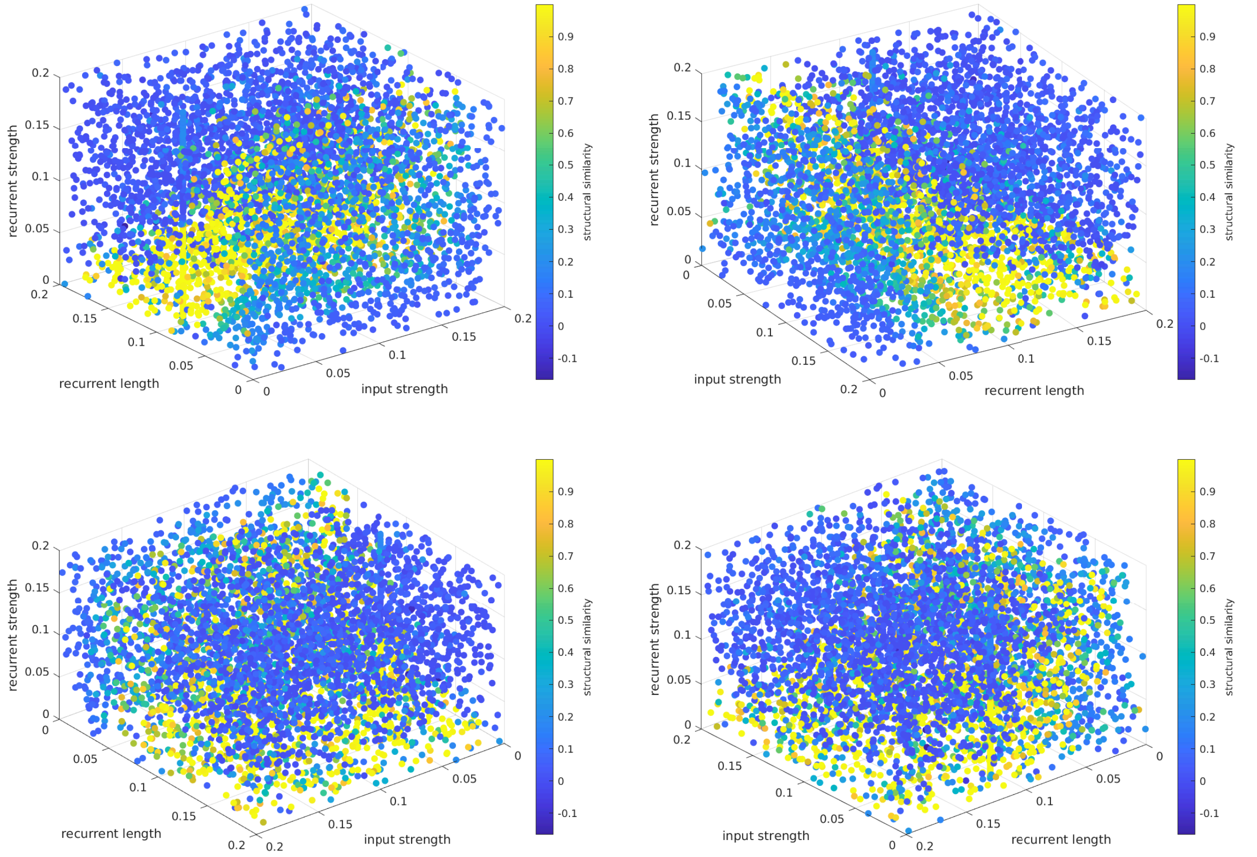

Supplementary Figure 1. Distribution of the total structural similarity (structural similarity of the entire movie) for the moving bump example (colour scale) throughout the parameter space (axes of recurrent strength, recurrent length, and input strength). Four views of the same parameter space shown. Source data are provided as a Source Data file.

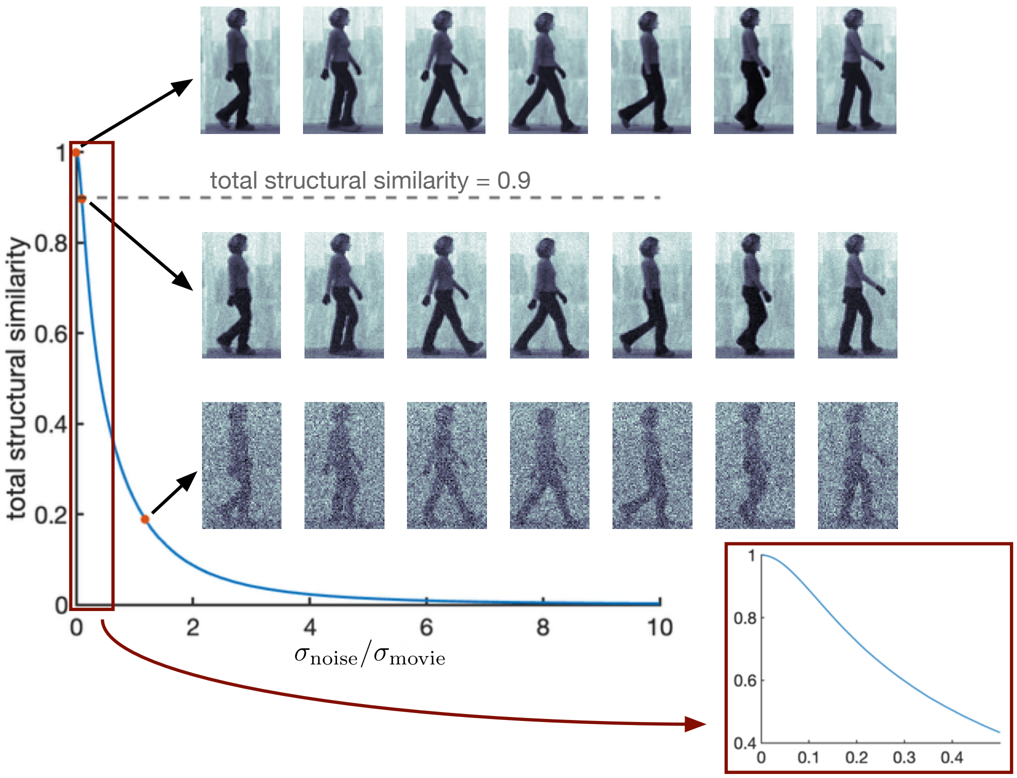

Supplementary Figure 2. Total structural similarity provides a sensitive measure of movie similarity. The total structural similarity between an example movie of a walking subject and the same movie with added Gaussian noise demonstrates how this measure captures similarity between movies. With progressively higher noise amplitude ($\sigma_{\text{noise}}/\sigma_{\text{movie}}$ in line plot), the total structural similarity rapidly decreases from 1 (no noise) to low values. Example image sequences are provided at different noise amplitudes (red dots on blue line) to illustrate the movie sequence at varying levels of added noise. Within a range of small noise amplitudes (inset), the total structural similarity drops rapidly from 1 to 0.4. Source data are provided as a Source Data file

Total SSIM	
phase-unshuffled	phase-shuffled
1.00	0.0126
	0.0143
	0.0086
	0.0125
	0.0095
	0.0109
	0.0211
	0.0018
	0.0069

Supplementary Table 1. Total structural similarity (SSIM) between ground-truth natural video and closed-loop forecast frames for regular case (left column) and case where each frame of the video is phase-shuffled using the discrete Fourier transform (right column). Shuffling was performed 10 times (mean $\pm$ standard deviation $= 0.0169 \pm 0.0050$).

input	reservoir size	video frame size (pixels)	# of training frames	training time (s)	closed-loop forecast performance (total SSIM)
moving bump	50x50	30x30	300	26.48	1.00
walking person	50x50	80x50	432	36.07	1.00

Supplementary Table 2. Training specifications for both forecasting examples for the cases of optimal hyperparameters. Total SSIM, total structural similarity.

total SSIM	
unshuffled	shuffled
0.9996	0.0186
0.9978	0.3692
0.9998	0.4609
0.9993	0.5982
0.9999	0.0126
0.9989	0.1804
0.9982	0.0091
0.9998	0.4609
0.9992	0.0653
0.9998	0.4553

Supplementary Table 3. Left column: a random sample of ten well performing (total SSIM > 0.99) network implementations (mean $\pm$ standard deviation $= 0.9988 \pm 0.0014$). Right column: topographically randomized versions of the network implementations, in which the recurrent weights and time delays were shuffled (mean $\pm$ standard deviation $= 0.2566 \pm 0.2186$). Total SSIM, total structural similarity.

Movie	$SSIM_{train}$	$SSIM_{test}$
Shahar walk	1.00	0.9999999254
Moshe walk	1.00	0.9999995867
Lyova walk	1.00	0.9999999899
Lena walk1	1.00	0.9999563115
Lena walk2	1.00	0.9999995154
Ira walk	1.00	0.9999872030
Ido walk	1.00	0.9999999976
Eli walk	1.00	0.9999993216
Denis walk	1.00	0.9999968517
Daria walk	1.00	0.9999962703

Supplementary Table 4. Results of 100 trials of the random-search optimization method performed on the free parameters for each walking movie in the Weizmann dataset, in terms of the total structural similarity (SSIM)-values during training and testing ($SSIM_{train}$ and $SSIM_{test}$, respectively) of the best trial for each movie.
