# Supplementary material for: Waves traveling over a map of visual space can ignite short-term predictions of sensory input
Source: Nat Commun. 2023 Jun 9;14:3409. doi: 10.1038/s41467-023-39076-2 (PMC10256723; doi:10.1038/s41467-023-39076-2)
Supplement: Supplementary file 2 — Description of Additional Supplementary Files [file 41467_2023_39076_MOESM2_ESM.pdf]

Description of Additional Supplementary Files

File Name: Supplementary Movie 1
Description: Closed-loop forecast of the moving bump. Left: ground-truth frames. Second from left: closed-loop forecast for the network with optimal recurrence. Second from right: network activity for the network with optimal recurrence. Right: closed-loop forecast for the network with no recurrence.

File Name: Supplementary Movie 2
Description: Closed-loop forecast of the natural movie. Left: ground-truth frames. Second from left: closed-loop forecast for the network with optimal recurrence. Second from right: network activity for the network with optimal recurrence. Right: closed-loop forecast for the network with no recurrence.

File Name: Supplementary Movie 3
Description: Closed-loop forecasts of topographic network model (top row) versus topographically shuffled network model (bottom row) in which the recurrent weights and time delays were shuffled.

File Name: Supplementary Movie 4
Description: Forecasts of the movie-switching example. Left: ground-truth frames. Right: forecasts.
